# Retrotransposon: a versatile player in human preimplantation development and health

**DOI:** 10.1093/lifemedi/lnac041

**Published:** 2022-09-25

**Authors:** Yuliang Qu, Zsuzsanna Izsvák, Jichang Wang

**Affiliations:** Department of histology and embryology, Zhongshan School of Medicine, Sun Yat-sen University, Guangzhou 510080, China; Key Laboratory for Stem Cells and Tissue Engineering (Sun Yat-sen University), Ministry of Education, Guangzhou 510080, China; Max Delbrück Center for Molecular Medicine in the Helmholtz Association, Berlin 13092, Germany; Department of histology and embryology, Zhongshan School of Medicine, Sun Yat-sen University, Guangzhou 510080, China; Key Laboratory for Stem Cells and Tissue Engineering (Sun Yat-sen University), Ministry of Education, Guangzhou 510080, China

More than half of human genome is comprised of transposable elements (TEs), most of which are the retrotransposons (or class I TEs). As the name suggests, retrotransposons propagate via an RNA intermediate that is reversely transcribed and integrated into new genomic loci (a so-called “copy and paste” mechanism). There are three main types of retrotransposons in human genome, including the long terminal repeats (LTRs), non-long terminal repeats (non-LTRs), and SINE-VNTR-Alu (SVA) elements. The LTR elements or endogenous retroviruses (ERVs) are derived from ancient retroviral infections. Over millions of years of evolution, multiple ERV-derived sequences have been fixed in vertebrate genomes, constituting around 8% of human genome. On the other hand, the non-LTRs, including long interspersed nuclear elements (LINEs) and short interspersed nuclear elements (SINEs), approximately account for 30% of human genome. The LINE-1 (L1) is an autonomous element with the ability to produce the whole enzymatic machinery for mobilization. In contrast, as one type of nonautonomous hominid-specific retrotransposons, SVA can be potentially mobilized in *trans* by hijacking the retrotransposition machinery produced by autonomous L1 elements. Despite their substantial occupancy of the human genome, most of TEs remain silenced, and have been long referred as “junk DNA” or “fossil records” of ancestral TE invasions. Recently, thanks to the advances in multiomics and genome-editing techniques, accumulating evidence suggests that the “junk DNA” could occasionally have a critical function for the human development and health.

## Retrotransposons in early human development

Following the evolution of the TEs, ERVs exhibit the original features of their ancestral proviruses to variable extents, ranging from full length elements to highly fragmented relics of the proviral genome. However, some elements occasionally get co-opted (domesticated or exapted) for a beneficial function for the host. For example, proteins encoded by HERVs might be repurposed to support human embryonic development. Such well-known examples are the retroviral envelope gene-coded syncytins (e.g., HERVW-1, HERV-FRD1), playing an essential role in placental syncytiotrophoblast formation. Furthermore, Rec, the accessory protein of the phylogenetically youngest HERVK (HML-2, human mouse mammary tumor virus like-2), was suggested to promote antiviral defense response in human preimplantation embryos [1]. Besides the full-length ERV sequences with partial coding potential, around 85% of human ERVs (HERVs) exist as solitary LTRs, termed “solo LTRs,” which provide a rich source of *cis*-­regulatory elements for gene expression during human embryonic development. Intriguingly, various primate/hominoid-specific retrotransposons are stage-specifically reactivated during human preimplantation development, indicating potential roles of these retrotransposons in rewiring regulatory network for early human development. They usually serve as binding platforms for multiple pluripotency transcription factors, forming a regulatory network for pluripotency [2]. Unexpectedly, LTR5_Hs elements, the human-specific regulatory sequences of HERVK, have been reported as functional enhancers that contribute to the specification of human primordial germ cells, suggesting the essential role of the phylogenetically youngest retrotransposons in human germline development [3].

**Figure 1.**
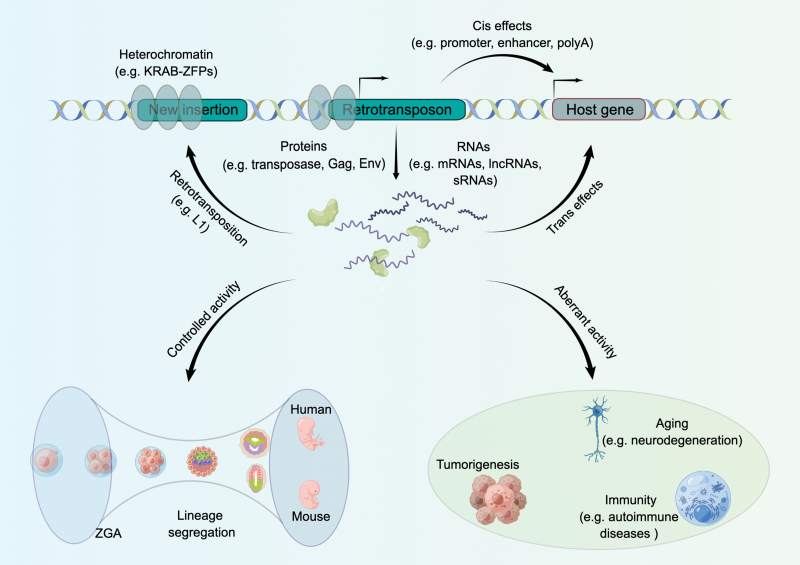
Retrotransposons in human development and disease. Under proper control of the surveillance system, retrotransposons could regulate gene expression in both *cis* and *trans*. Functionally, human development might benefit from retrotransposons that exert multiple important roles in early embryogenesis, including zygote genome activation (ZGA) and lineage segregation in preimplantation embryos. On the other hand, aberrant expression of retrotransposons has been implicated in various pathological processes, such as tumorigenesis, autoimmune diseases, and aging. The figure was prepared by Figdraw.

Importantly, the stage-specific activation of retrotransposons depends on precise epigenetic regulation during early human development [2, 4]. Recently, exploiting low-input ChIP-seq techniques, Xu *et al.* and Yu *et al.* independently discovered the significance of the stage-specific TE associated H3K9me3 landscape in human preimplantation embryos [2, 4]. In early human embryos, similar to mice, specific Krüppel-associated box domain zinc finger proteins (KRAB-ZNFs) have an essential role in regulating the transcription of different LTR families via stage-specific deposition of H3K9me3 [4]. During the transition from 2-cell to the 8-cell (8C) stage of human embryos, the decreased deposition of H3K9me3 on the ­hominoid-specific SVA_D retrotransposons facilitates the interaction of these elements with zygotic genome activation (ZGA) genes [2]. Intriguingly, a set of SVA_D elements could function as enhancers to promote the human ZGA by providing binding sites for 8C transcription factors (e.g., DUXA and ZSCAN4) [2]. Of note, CRISPR-KRAB-mediated silencing of SVA_D elements led to defective ZGA and compromised blastocyst formation, demonstrating the crucial role of the SVA-derived enhancers in early human development [2]. Moreover, *de novo* deposition of H3K9me3 in trophectoderm prevents binding of pluripotency transcription factors to certain hominoid-specific retrotransposons which function as regulatory elements for ICM-specific genes, suggesting potential roles of these *de novo* H3K9me3 domains in lineage segregation in preimplantation embryos [2, 4]. It will be of interest to explore whether retrotransposons also contribute to species-specific regulation for lineage specification in postimplantation embryos (Fig. 1).

## Retrotransposons in human diseases

Despite their potential functionalities in development, TEs remain strictly controlled by human genomic surveillance system in somatic cells, but can be dysregulated in pathological conditions or aging. As one human-specific ERV, aberrantly transcribed HERVK (HML-2) can activate the innate immune response to tumors and aging as well as viral infection, indicating complex roles of retrotransposons in pathological conditions, maybe, in a species-specific manner. The role of HERVK (HML-2) in neurodevelopment is particularly intriguing. Using human pluripotent stem cell-based model combined with the CRISPR-VPR system, Padmanabhan Nair *et al.* recently reported that aberrant reactivation of HERVK (HML-2) might impair cortical neuronal development by inducing premature expression of genes involved in neuronal differentiation [5], implying that aberrant reactivation of HERVK may be implicated in various neurological disorders including neurodegenerative diseases. Most intriguingly, similar to that in human blastocysts [1], multiple copies of the phylogenetically young HERVK (HML-2) have intact open reading frames and hold the potential to produce retrovirus-like particles (RVLPs) in various pathological conditions as well as senescent cells [6]. However, the function of HERVK-derived RVLPs in tumor progression and aging remains largely unknown. It is interesting to explore whether and how HERVK-derived RVLPs regulate immune response to cancer and aging in the future.

LINE-1 (L1) was shown to affect the function of immune cells, which might also play crucial roles in immune response to cancer and aging. Liang *et al.* recently revealed that BMAL1, an essential component of the molecular circadian clock, could prevent stem cell senescence via stabilizing heterochromatin and thus repressing activation of the L1-cGAS-STING pathway, highlighting that the aberrantly activated L1 transcription drives cellular senescence by stimulating IFN-I-directed innate immune signaling [7]. Unexpectedly, the transcripts containing L1-derived sequences might contribute to maintaining the quiescent state of naive CD4+ T cells, whereas these transcripts are downregulated upon T cell activation [8]. Expression of “L1-containing transcripts” was also observed in dysfunctional T cells, suggesting their potential function in governing T cell exhaustion [8].

Importantly, as the only autonomously mobile transposons in human, L1_Hs may induce insertional mutagenesis. Indeed, pan-cancer genomic analyses revealed that nearly half of human cancer cell lines contain somatic retrotransposition events, suggesting the relevant role of L1 insertion in some human oncogenesis by causing megabase-scale deletions [9]. However, one recent study shows the potential role of L1 in repressing tumor progression. The methyl H3K9-binding protein MPP8, a component of the HUSH complex, was shown to suppress L1 in myeloid leukemia, whereas the reactivation of L1 impairs leukemia cell growth *in vitro* and in xenotransplants, suggesting tumor-type-specificity of L1 retrotransposition [10].

Altogether, these recent studies support the notion that co-option of retrotransposons might contribute to human development. On the other hand, the frequently reported dysregulation of retrotransposons in pathological conditions underlines the significance of the genome surveillance system (Fig. 1). In fact, like other eukaryotes, humans have evolved a panel of highly orchestrated molecular mechanisms to control TE activity, including the PIWI/piRNA machinery, KRAB-ZFP-KAP1 complex, DNA methylation, histone modifications, inhibition of splicing, and RNA modifications. Given the impact of TEs in human aging, neurodegeneration, inflammation, and cancer, it is of particular tempt to investigate the underlying mechanisms that keep them under control. Furthermore, despite the advances, how those TE-derived sequences have been co-opted in the human genome remains to be elucidated. Recent progresses in gene editing technology and long-read sequencing would pave the way to further decipher versatile roles of these elements in human embryonic development and diseases.
